# Does a stronger moral identity lead to a more reserved sense of humor? The influence of moral identity on sense of humor and its underlying psychological mechanisms

**DOI:** 10.1002/pchj.797

**Published:** 2024-09-16

**Authors:** Liting Fan, Binghai Sun, Shuwei Lin, Jiahao Zhou, Tenglong Chen

**Affiliations:** ^1^ School of Psychology Zhejiang Normal University Jinhua China; ^2^ Zhejiang Philosophy and Social Science Laboratory for the Mental Health and Crisis Intervention of Children and Adolescents Zhejiang Normal University Jinhua China

**Keywords:** benign violation theory, humor appreciation, humor sharing, moral identity, social distance

## Abstract

Three studies were conducted to examine the influence of moral identity on sense of humor, employing the benign violation theory (BVT) as a theoretical framework. Study 1 (*n* = 350), a questionnaire‐based survey, aimed to establish a preliminary exploration of the relationship between moral identity and sense of humor. Studies 2 (*n* = 172) and 3 (*n* = 172) jointly examined the impact of activated moral identity on sense of humor (humor appreciation, humor sharing) through the recollection and writing task. The results of these studies indicated that the effects of (activated) moral identity on the sense of humor (humor appreciation, humor sharing) were moderated by the type of humor and social distance of the target. On the one hand, high levels of (or activated) moral identity can significantly and positively predict sense of humor (humor appreciation, sharing); on the other hand, when there is a moral violation in the humor, and the target involved is at a close social distance, activated moral identity decreases the humor appreciation and humor sharing, where benign judgment plays a mediating role. These findings enrich the understanding of the complex relationship between moral identity and sense of humor, and have significant theoretical and practical implications.

## INTRODUCTION

Humor pervades all aspects of life (Stieger et al., 2011). A sense of humor serves as a facilitator for interpersonal communication, promoting smooth interactions (Cao, Hou, et al., 2023a; Cao, Liu, et al., 2023b). Skillful utilization of humor can effectively mitigate interpersonal conflicts (Young, 2008), foster trust among individuals (Bippus et al., 2012), and enhance satisfaction with interpersonal exchanges (Chiew et al., 2018). Furthermore, an individual's sense of humor is often positively associated with their physical and mental well‐being (Herzog & Strevey, 2008). These findings underscore the importance of investigating the role of sense of humor in both interpersonal dynamics and personal growth. However, an individual's inclination to express humor during social interactions may be influenced by their moral identity. The present research aimed to investigate the influence of moral identity on sense of humor (humor appreciation, humor sharing). Additionally, this research endeavored to uncover the underlying psychological mechanisms involved.

### Moral identity, humor appreciation, and humor sharing

Humor appreciation and humor sharing are indicators of an individual's psychological and behavioral inclinations, reflecting that individual's attitudes toward a particular subject matter. Specifically, humor appreciation refers to the degree to which an individual perceives a humorous stimulus as amusing when exposed to it (Cao, Hou, et al., 2023a; Warren et al., 2021). Additionally, humor sharing refers to the inclination to disseminate humor among others (Cao, Hou, et al., 2023a). According to the humor level theory, humor appreciation is a prerequisite for humor sharing, and individuals are more likely to share a certain type of humor with others to facilitate interpersonal interactions when they have an attitudinal appreciation toward it (Zhang, 2015). Martin et al. (2003) categorized humor into four distinct styles: affiliative, self‐enhancing, aggressive, and self‐defeating. Affiliative humor refers to the expression of humor in a friendly manner; self‐enhancing humor reflects the resilience where individuals use humor as a motivational force in challenging situations; aggressive humor involves a derogatory approach, seeking amusement at the expense of others; and self‐defeating humor is characterized by the use of self‐derogation to amuse others. Martin et al. (2003) posited that aggressive humor and self‐defeating humor are nonadaptive forms that impede the development of an individual's mental well‐being, as they involve ridiculing others or oneself for personal gratification. In contrast, affiliative and self‐enhancing humor are adaptive forms that foster the development of an individual's mental health by employing humor in a positive and optimistic manner. Furthermore, nonadaptive humor exhibits a negative orientation in terms of definitions and scale questions, encompassing numerous moral transgressions; whereas adaptive humor demonstrates a positive orientation devoid of moral violations.

Moral identity is a self‐concept organized around a set of moral traits such as integrity, honesty, and sincerity, reflecting the degree of congruence between self‐values and societal values (Aquino & Reed, 2002; Reed & Aquino, 2003). Mulder and Aquino (2013) proposed that moral identity, as an integral part of the self‐regulation mechanism in the moral domain, exerts significant influence on an individual's behavioral performance. Specifically, when an individual's moral identity is activated, they engage in a self‐evaluation process by comparing their current moral self‐image with the activated moral identity. If a discrepancy exists between the two, it may lead to psychological distress for the individual. To avoid perceiving themselves as immoral or being perceived as such by others, individuals under such stress tend to reject immoral behavior and project a positive moral image. Numerous empirical studies have demonstrated that moral identity is significantly and negatively associated with various forms of immoral behaviors, including aggression, antisocial conduct, and bullying (Hardy et al., 2014; Kavussanu & Ring, 2017).

Individuals with a strong moral identity are perceived as trustworthy (X. Chen et al., 2021), while those exhibiting a sense of humor tend to be well‐liked (Chiew et al., 2018); both attributes are fundamental components of personal charisma. People with a strong moral identity are likely to uphold and enforce social rules and norms of behavior to a significant degree (Mulder & Aquino, 2013). It can be argued that individuals with a strong moral identity effectively adhere to societal moral standards by refraining from engaging in behaviors that contradict their ethical principles. However, it is worth noting that those who prioritize morality may be perceived as stereotypical and inflexible because of their unwavering commitment to a moral code. Simultaneously, individuals with a sense of humor exhibit proficiency in challenging conventional concepts and norms, forging connections between typically unrelated elements, and demonstrating a high level of ingenuity (C. H. Chen et al., 2019; Y. Zeng, 2013). From this perspective, there may be a negative correlation between the moral identity demonstrated by individuals and their sense of humor.

However, previous research on the influence of moral identity on sense of humor has yielded inconsistent results. Upon reviewing the existing literature, we have found that this inconsistency is likely related to the moral violations involved in the jokes. Some studies have shown that activated moral identity reduces an individual's sense of humor (Paramita et al., 2022; Yam et al., 2019). Yam et al. (2019) examined the impact of moral identity and joke type on humor appreciation. The findings revealed that when exposed to jokes containing moral violations, the group with activated moral identity perceived the jokes as less humorous compared with the control group; however, in the case of jokes lacking moral violations, there was no significant difference observed in humor appreciation between the two groups. Another study has also examined the effect of moral identity on evaluations of humorous advertisements (Paramita et al., 2022). The research suggested that activated moral identity induces a decline in positive evaluations of humorous advertisements with harm‐violation by eliciting an increased sense of aversion. According to Yam et al. (2019), appreciating, sharing, and creating humor involving moral violations can be considered ethically questionable. Activation of moral identity elicits individuals' aversive responses to both engaging in and contemplating immoral actions, thereby leading individuals to exhibit greater resistance toward such behaviors. Individuals with activated moral identity (compared with the control group) exhibit heightened sensitivity toward moral violations, and demonstrate a reduced tendency to perceive content as benign (Hardy & Carlo, 2011; Paramita et al., 2022), consequently influencing their humor appreciation. However, the activation of moral identity does not necessarily diminish an individual's sense of humor. Although activated moral identity reduced positive evaluations of humorous advertisements with harm‐violation compared with the control group, there was no significant difference in humor appreciation (Paramita et al., 2022). In response to the discrepancies in the existing research findings, we will now proceed to discuss the matter from the perspective of the benign violation theory (BVT).

### The benign violation theory of humor

The concept of BVT originated from Veatch's (1998) hypothesis that humor arises from benign violations. McGraw and his colleagues (2010, 2012, 2014) supported the hypothesis through a series of empirical studies and formalized the BVT of humor. BVT emphasizes that a sense of humor emerges from moderate violations. According to McGraw, a stimulus must possess both violation and benign qualities to elicit laughter. Specifically, a violation can pertain to physical, moral, physiological, or common‐sense norms. Benign implies that the degree of violation is within an acceptable range, not perceived as excessively offensive by individuals. However, determining the boundaries of this benign quality is challenging.

Hodson et al. (2010) proposed that the inclusion of cavalier humor beliefs (CHB) enhances our comprehension of the concept of “benign” within humor. CHB represents the perspective of individuals who approach jokes with a lighthearted disposition, embodying the belief that humor should be regarded solely as jests (Hodson et al., 2010; L. J. Li & Wang, 2022). Individuals exhibit elevated levels of CHB when they perceive a morally offensive joke as intended solely for entertainment, without engaging in critical evaluation of its offensiveness. Due to perceiving a joke as merely comical and considering the moral transgression of the joke as inconsequential, these individuals exhibit an increased level of appreciation for such humor (Buie et al., 2022). However, Hodson et al. (2010) observed that CHB served as a significant positive predictor of humor appreciation specifically for jokes involving moral violations, not for nonmorally violating jokes. According to the BVT, a joke is considered naturally benign if it deviates from conventional thinking without violating moral standards. Furthermore, empirical research has demonstrated that women with high levels of CHB perceive less aggression in response to jokes that are demeaning and offensive toward women, while simultaneously exhibiting a greater appreciation for such humor (Prusaczyk & Hodson, 2020).

Furthermore, research comparing Eastern and Western cultures has indicated that in the context of Eastern cultures, particularly in China, people's understanding of morality (and immorality) and humor possesses unique characteristics (Buchtel et al., 2015; Buchtel et al., 2022; Cao & Hou, 2023; Qian, 2007). Buchtel et al. (2015) suggested that in Western cultural contexts, “immoral” is understood as “harmful,” while in Eastern cultural contexts, “immoral” is perceived as “uncivilized.” Therefore, in terms of the degree of immoral transgression, Eastern cultures are relatively less intense than Western cultures. Additionally, humor in China has historically endured a protracted period of being undervalued and marginalized within the context of mainstream culture. Traditional Confucian values, which have long been considered orthodox, emphasized moral literature and sought to integrate morality into every aspect of people's lives, perceiving humor as crude and vulgar (Qian, 2007). However, in contemporary society, humor has gained recognition as a charismatic attribute. It is widely acknowledged among college students that humor is significant for both personal development and societal well‐being. Yet, a comprehensive understanding of their own sense of humor remains scarce (Qian, 2007).

As a matter of fact, some individuals exhibit a heightened level of moral identity alongside a pronounced sense of humor. Even in ancient times, when social norms were stringent, Confucius—known for his unwavering adherence to moral principles—frequently displayed humor in both his words and actions (Lai, 2007). As we mentioned earlier, “having a sense of humor” is a positive evaluation of a person. To a large extent, a sense of humor is an influencing factor in interpersonal attraction and is largely considered a manifestation of “wisdom” (Cao, Liu, et al., 2023b). In numerous psychological studies, a sense of humor is often associated with creativity (C. H. Chen et al., 2019). On the contrary, in stereotypes, an individual perceived as morally upright may also be seen as rigid and stubborn, which can be, to a certain extent, detrimental to their social development. Therefore, from a practical standpoint, they might actually benefit from exhibiting more humor. In this case, appreciating adaptive humor (or nonmoral violation jokes) is a straightforward and relatively easy endeavor. A meta‐analysis of the relationship between humor and the Big Five personality traits found adaptive humor to be positively correlated with agreeableness, conscientiousness, and openness (Plessen et al., 2020). Similarly, individuals with a high level of moral identity also exhibit higher levels of agreeableness, conscientiousness, and openness (C. Chen & Wang, 2023). They often demonstrate prosocial behavior in social interactions, so when it comes to humor that does not violate moral principles, individuals with a high level of moral identity may, surprisingly, show a high tolerance for “deviance,” exhibiting a greater appreciation for adaptive humor (or nonmoral violation jokes). Therefore, it is imperative to conduct further research on the correlation between moral identity and sense of humor within the contemporary social context.

### The role of social distance

Humor can sometimes serve as an indicator of intellectual, educational, and moral superiority (J. Liu, 2016). From this perspective, it is plausible that moral identity and sense of humor may not be in competition. In terms of form, joke‐telling represents an attempt to rationalize the inherent irrationality of humor, often downplaying the component involving moral violation (Freud, 1928). Freud (1928) posited that while jokes may contain moral violations in their content, their purpose is not to harm others. Thus, the enjoyment and sharing of such jokes are not inherently considered immoral behaviors. In other words, high levels of moral identity might require the consideration of additional moderating variables to understand how they could potentially reduce the sense of humor about jokes containing moral violations.

The impact of social distance on individuals' sense of humor depends on variations in their tendencies for selective information processing. Social distance refers to a subjective perception of others being either in proximity or at a distance from oneself (Ross & Wilson, 2002; Van Boven et al., 2010). Specifically, individuals who are not familiar with each other exhibit greater social distance than those with an established friendship. Previous studies have shown a positive correlation between social distance and the level of humor appreciation, suggesting that humor is perceived as more amusing as social distance increases (McGraw et al., 2012, 2014). According to the construal level theory, individuals are more likely to adopt a generalized and abstract approach when making judgments about information they perceive as being at a greater social distance (Kan, 2010; Zhong & Chen, 2013). Specifically, when a joke is directed toward an unfamiliar individual, the recipient tends to disregard the target of the joke and focuses on the content's specifics, such as its form and outcome, leading to enjoyment. However, when the target of the joke is within a close social distance, recipients engage in more specific processing, focusing on the target's character and the purpose of the event. This focused processing can diminish the amusement derived from the joke, reflecting an underlying “non‐benign” effect. When a joke involves a moral violation, individuals perceive a greater sense of threat if the social distance is close, leading to decreased acceptance of such jokes. Conversely, as social distance increases, individuals perceive less threat and are more likely to accept and enjoy these jokes (McGraw et al., 2012). There are also researchers who have found, based on corpus and empirical studies, that the majority of humorous content features targets at a distance rather than those at a close distance (Yamane et al., 2021).

Additionally, individuals with elevated levels of moral identity exhibit cognitive processing that emphasizes moral considerations more significantly, thereby placing a higher value on the moral self‐fulfillment and demonstrating heightened moral prudence (Forehand et al., 2002). The proximity of social distance can amplify the moral implications of an event, thereby intensifying the perception of immorality for those involved (Zheng, 2008). When the social distance is close, the moral character of a situation becomes more pronounced, leading individuals with high levels of moral identity to focus more on the moral components. Consequently, jokes are more likely to be perceived as non‐benign, which subsequently influences humor appreciation and sharing. Conversely, when the social distance is more distant, the moral character may be disregarded, and even individuals with high levels of moral identity might not readily focus on the moral components within a short timeframe. As a result, their moral identity might not significantly impact the judgment of benignity.

Building on previous research, the present study anticipates that social distance will significantly influence individuals' sense of humor. Moreover, it is expected to potentially serve as a moderator in the relationship between moral identity and sense of humor.

### The current research

Drawing upon the literature review and the BVT framework, we hypothesized that the effect of moral identity on sense of humor would be influenced by the joke type and social distance. Specifically, we anticipated a negative association between moral identity and the sense of humor related to moral violations, while expecting a positive association with humor unrelated to moral violations. Simultaneously, we hypothesized that the influence of moral identity on sense of humor would be moderated by social distance, particularly when individuals are exposed to jokes involving moral violations. That is, the impact of moral identity on reducing sense of humor is expected to be more pronounced when the target of the jokes is someone with whom the individual has a close social relationship, as opposed to someone with whom they have a greater social distance. We propose that this effect is mediated by a reduction in benign evaluations (i.e., CHB). The present research was conducted to test these hypotheses using a sample of college students. Study 1 utilized the self‐importance of moral identity scale and a humor style questionnaire to preliminarily investigate the relationship between moral identity and sense of humor. Study 2 examined the interaction effect of moral identity and joke type on humor appreciation and sharing by manipulating moral identity. Study 3 further explored the interaction effect of moral identity and social distance on humor appreciation and sharing in jokes involving moral violations, by manipulating the moral identity, while also investigating the mediating role of benign evaluations (CHB).

## STUDY 1

Study 1 used the self‐importance of moral identity scale and the humor style questionnaire for college students to conduct a preliminary examination of the relationship between moral identity and sense of humor through a questionnaire survey.

### Method

#### Participants and procedures

Study 1 released the questionnaires through an online platform (a professional online questionnaire platform in China, https://www.wjx.cn/) and Chinese university students were recruited to participate for the survey.

A total of 427 college students engaged with the questionnaire, which included sections on basic personal information, the self‐importance of moral identity scale, and the humor style questionnaire. Data from 77 participants who failed all reverse‐scored items were excluded, yielding a final sample size of 350 valid responses (148 males and 202 females; *M*_age_ = 19.55, *SD* = 2.17).

#### Measure

##### Self‐importance of moral identity scale

*The self‐importance of moral identity scale* developed by Aquino and Reed (2002) was utilized to assess participants' moral identity. The scale comprises two dimensions: internalization (5 items, e.g., “It would make me feel good to be a person who has these characteristics”, with 2 reverse‐coded items) and symbolization (5 items, e.g., “I often wear clothes that identify me as having these characteristics”). Each item was rated on a 7‐point Likert scale, ranging from 1 (*completely disagree*) to 7 (*completely agree*), with higher mean scores indicating higher levels of moral identity. In Study 1, Cronbach's α for the total scale was 0.84, for the internalized dimension was 0.76, and for the symbolized dimension was 0.82.

##### Humor style questionnaire

In Study 1, *the humor style questionnaire*, adapted by G. Chen and Martin (2007) from the original work of Martin et al. (2003), was selected for use. The scale consists of 25 items across four dimensions: affiliative humor (8 items, e.g., “I don't have to work very hard at making other people laugh—I seem to be a naturally humorous person”, with 5 reverse‐coded items), self‐enhancing humor (5 items, e.g., “If I am feeling depressed, I can usually cheer myself up with humor”), aggressive humor (7 items, e.g., “Sometimes I think of something that is so funny that I can't stop myself from saying it, even if it is not appropriate for the situation”), and self‐defeating humor (5 items, e.g., “I will often get carried away in putting myself down if it makes my family or friends laugh”). In this study, a 7‐point Likert scale was employed, and the mean score was calculated for each dimension separately, ranging from 1 (*completely disagree*) to 7 (*completely agree*), with a higher mean score indicating a higher sense of humor. In Study 1, the Cronbach's α for affiliative humor, self‐enhancing humor, aggressive humor, and self‐defeating humor were 0.74, 0.77, 0.89, and 0.84, respectively.

### Results and discussion

#### Test of common method biases

Study 1, being a questionnaire‐based survey, was susceptible to potential common method biases. To address this, all self‐assessment items were tested for common method bias through validated factor analysis. The results indicated a poor model fit for Study 1 (*χ* ^2^/df = 6.32、CFI = 0.47、GFI = 0.50、AGFI = 0.44、NFI = 0.43、RMSEA = 0.12), suggesting that there was no significant common method bias present (L. Liu et al., 2019).

#### Descriptive statistics and regression analysis

The results of the descriptive statistical analysis of the variables are presented in Table 1. Study 1 revealed that moral identity had significant positive correlations with adaptive humor (*r*_affiliative_ = 0.34, *p* < .001; *r*_self‐enhancing_ = 0.33, *p* < .001), and significant negative correlations with nonadaptive humor (*r*_aggressive_ = −0.15, *p* = .005; *r*_self‐defeating_ = −0.12, *p* = .030). Further regression analyses, with moral identity as the predictor variable and various humor styles as outcome variables, indicated that individuals' moral identity positively predicted their adaptive humor (*β*_affiliative_ = 0.36, *t* = 6.65, *p* < .001; *β*_self‐enhancing_ = 0.43, *t* = 6.50, *p* < .001), and negatively predicted their nonadaptive humor (*β*_aggressive_ = −0.22, *t* = −2.85, *p* = .005; *β*_self‐defeating_ = −0.17, *t* = −2.18, *p* = .030).

**TABLE 1:** Correlation coefficients of variables for Study 1 (*n* = 350)

	*M ± SD*	1	2	3	4	5	6	7
1. Moral identity	5.33 *±* 0.94	‐						
2. Internalization	5.79 *±* 1.03	0.85***	‐					
3. Symbolization	4.87 *±* 1.15	0.88***	0.50***	‐				
4. Affiliative	4.63 *±* 1.01	0.34***	0.31***	0.28**	‐			
5. Self‐enhancing	4.47 *±* 1.24	0.33***	0.22***	0.34***	0.28**	‐		
6. Aggressive	2.75 *±* 1.35	−0.15**	−0.26***	−0.02	−0.31**	0.14*	‐	
7. Self‐defeating	2.88 *±* 1.36	−0.12*	−0.22***	0.01	−0.37**	0.17**	0.77***	‐

* *p* < .05;

** *p* < .01;

*** *p* < .001.

Study 1 demonstrated that the effect of moral identity on sense of humor is influenced by the humor style, thus supporting the initial hypothesis of this research. Study 2 aimed to examine the moderating role of joke type in the relationship between moral identity and humor appreciation and sharing under controlled laboratory conditions.

## STUDY 2

### Method

#### Design and participants

Study 2 employed a 2 × 2 mixed design with a between‐subjects variable of moral identity (activation, control), a within‐subjects variable of joke type (moral violation, nonmoral violation), and the dependent variables were humor appreciation and sharing. The study aimed to examine whether participants' appreciation and sharing of humor varied across states of moral identity when exposed to different joke types. Using G*Power 3.1 (Faul et al., 2007), we conducted a priori power analysis, calculating that at least 34 participants would be required to detect effects with 80% power at a 0.05 significance level, given an expected effect size = 0.25. Guided by related studies (Cao, Liu, et al., 2023b; Paramita et al., 2022), we recruited 184 college students to ensure robust results in Study 2. After excluding 12 participants who either failed the recollection and writing task, did not maintain a serious attitude during the experiment, or did not complete the experimental process, a total of 172 participants (100 females, 72 males; age range of 17–28 years; *M*_age_ = 20.13, *SD*_age_ = 2.04) were included in the final analysis. Participants were promised that all information would be used solely for research purposes. Upon completion of the study, each participant received a payment of 10 RMB.

#### Material and procedure

##### Basic personal information

**FIGURE 1:**
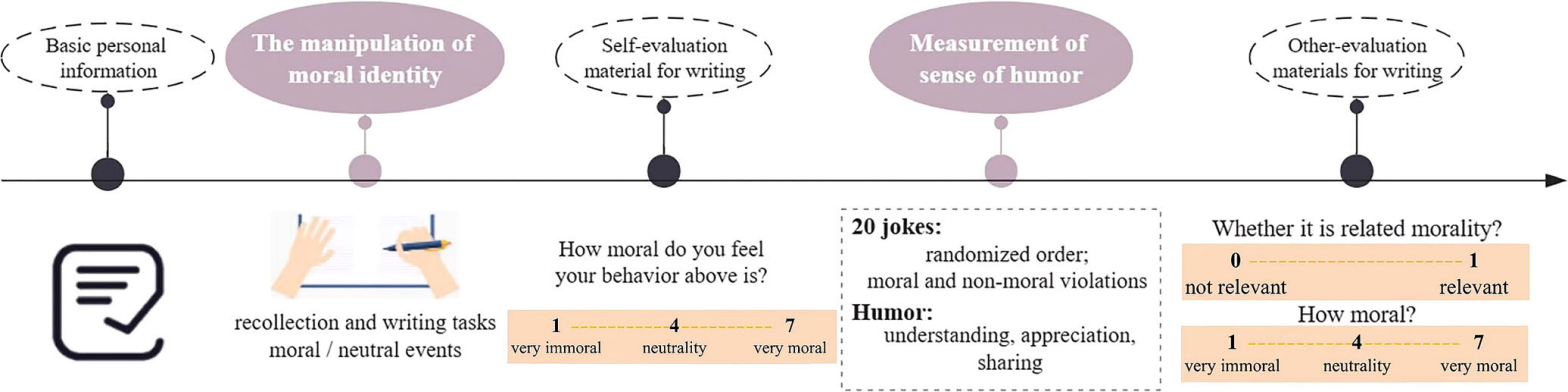
Flowchart for Study 2.

Basic personal information of the participants, such as name, gender, age, and profession, was first collected (see Figure 1 for the procedure).

##### The manipulation of moral identity

Participants were randomly assigned to either an activation or control group for moral identity. The manipulation of moral identity was achieved through recollection and writing tasks (Aquino et al., 2009; Yam et al., 2019). Those in the moral identity activation group were instructed to recall a morally significant event they had recently done and to write about it in the first person in as much detail as possible (approximately 200 words). Conversely, participants in the moral identity control group were asked to recall an event they had done yesterday, with all other requirements being consistent with those of the activation group.

Upon completing the writing task, participants were also asked to rate their behavior on a 7‐point scale as a subjective operational check, that is, “How moral do you feel your behavior described above is?” (1 = very immoral; 7 = very moral).

To ensure the validity of the moral identity manipulation, and in addition to self‐ratings by participants, five additional graduate students trained in psychology served as coders and assessed all participants' writing materials at the end of the entire experiment. Coders evaluated each submission based on two criteria: “whether the behavior in the material is morally relevant” (0 = *not relevant*; 1 = *relevant*) and “how moral is the behavior in the material” (1 =* very immoral*, 7 =* very moral*). If a participant in the activation group wrote about an event deemed by all coders to be morally irrelevant, or if a participant in the control group wrote about an event deemed morally relevant, that participant's data were excluded from subsequent analysis.

##### Measurement of humor appreciation and sharing

Different types of joke materials. We believe that jokes and moral violation may vary across cultures (Buchtel et al., 2015). Therefore, the jokes used in this study were selected by referencing previous research (Yam et al., 2019) and through an online corpus, resulting in a collection of 20 jokes—10 involving moral violations and the other 10 without. The jokes were evaluated by 39 psychology graduate students (*M*_age_ = 22.69, *SD* = 1.64) unrelated to the present study, who assessed the immorality and humor of the materials on a 7‐point scale (1 = *not immoral/not funny at all*, 7 = *very immoral/very funny*). The assessment indicated that jokes with moral violations were rated as more immoral than those without (*M*_moral violation_ = 3.07, *SD* = 1.29; *M*_nonmoral violation_ = 1.95, *SD* = 1.00, *t* = 6.88, *p* < .001, Cohen's *d* = 0.97). However, there was no significant difference in perceived funniness between the two types of jokes (*M*_moral violation_ = 3.89, *SD* = 1.52; *M*_nonmoral violation_ = 3.90, *SD* = 1.50, *t* = −0.19, *p* = .850, Cohen's *d* = 0.01). The above suggested that the selected jokes are suitable for this experiment.

The 20 jokes were presented to participants in a randomized order. After each joke, participants were asked to answer four questions:

- do you understand the punch line of the joke (1 = *not at all*; 7 = *completely understandable*);
- which line you found to be the punchline (participant indicates what he thinks is the punch line);
- how funny you found the joke (1 = *not funny* to 7 = *very funny*);
- would you like to share the joke with others (1 = *not at all willing* to 7 = *very willing*).

Questions (i) and (ii) served as additional variables to ensure comprehension of the jokes, as understanding humor is foundational to an individual's sense of humor (Zhang, 2015). In other words, whether or not a joke is funny and whether or not you want to share it with others is based on the fact that the individual understands the joke. Items (iii) and (iv) correspond to measures of humor appreciation and humor sharing, respectively (Cao, Hou, et al., 2023a).

### Results and discussion

#### The validity tests of the manipulation of moral identity

First, coded data were analyzed for five coders. According to the first item, Kendall's *W* for the coders' ratings was 0.80 (*p* < .001), indicating a strong agreement among the coders. Moreover, the coders identified three participants in the control group whose recollections involved moral behaviors, and nine participants in the activation group whose recollections were not morally related. Consequently, the data from these 12 participants were excluded from subsequent analyses.

An independent samples *t‐*test based on the coders' ratings for the second item revealed that the activation group (*M* = 5.36, *SD* = 0.55) had significantly higher moral scores than the control group (*M* = 4.08, *SD* = .19), *t* = 20.38, *p* < .001, Cohen's *d* = 3.11. Subsequently, another independent samples *t*‐test was conducted using the self‐rated counts of the 172 participants as the dependent variable. The results indicated that the activation group (*M* = 5.71, *SD* = 0.80) had significantly higher moral scores than the control group (*M* = 4.71, *SD* = 1.35), *t* = 5.94, *p* < .001, Cohen's *d* = 0.90. These findings suggest that the manipulation of moral identity was effective.

#### The validity of humorous material from the perspective of humor understanding

In this study, we selected jokes that fit into the local Chinese culture. Previous research has established that individuals' understanding of humor is fundamental to their sense of humor (Zhang, 2015). It suggests that an individual's assessment of a joke's funniness and their willingness to share it hinge on their ability to comprehend the joke. Consequently, this study needed to provide some assurance that participants not only read but also cognitively processed and understood the punchlines of the jokes presented.

We initially examined participants' subjective humor comprehension scores for a 7‐point Likert scale question, “*do you understand the punch line of the joke*?” The results indicated no significant difference in humor comprehension between the activation group and the control group of moral identity. Moreover, the mean humor comprehension scores for participants in both groups exceeded the theoretical midpoint of 4 (see Table 2), indicating a high level of humor comprehension without significant intergroup differences. Furthermore, the percentage of participants who correctly identified the punchline for each joke was derived based on where they pointed out the punchline, as shown in Table 3. Collectively, participants demonstrated a high accuracy rate in humor comprehension for each joke. These results further corroborate that the joke materials were appropriately selected and remained within the cognitive comprehension range of the individuals.

**TABLE 2:** Participants' humor understanding on two joke types under moral identity manipulation.

	Control group for moral identity (*M* ± *SD*)	Activation group for moral identity (*M* ± *SD*)	*t*	*p*
Moral violation	5.62 ± 1.26	5.65 ± 1.21	0.15	.879
Nonmoral violation	5.39 ± 1.29	5.64 ± 1.18	1.35	.180
Average	5.51 ± 1.22	5.64 ± 1.14	0.78	.435

**TABLE 3:** Correctness of punchline recognition for each joke.

	J1	J2	J3	J4	J5	J6	J7	J8	J9	J10
Moral violation	94.77%	99.84%	93.61%	94.19%	95.35%	81.40%	96.51%	96.51%	94.19%	90.70%
Nonmoral violation	91.86%	97.09%	94.77%	93.61%	97.67%	94.19%	87.21%	96.51%	94.77%	95.35%

*Note*: J1–J10 refer to the 10 jokes with moral violations and the 10 jokes with nonmoral violations, respectively.

#### The effect of moral identity and moral violation components on humor appreciation and sharing

**FIGURE 2:**
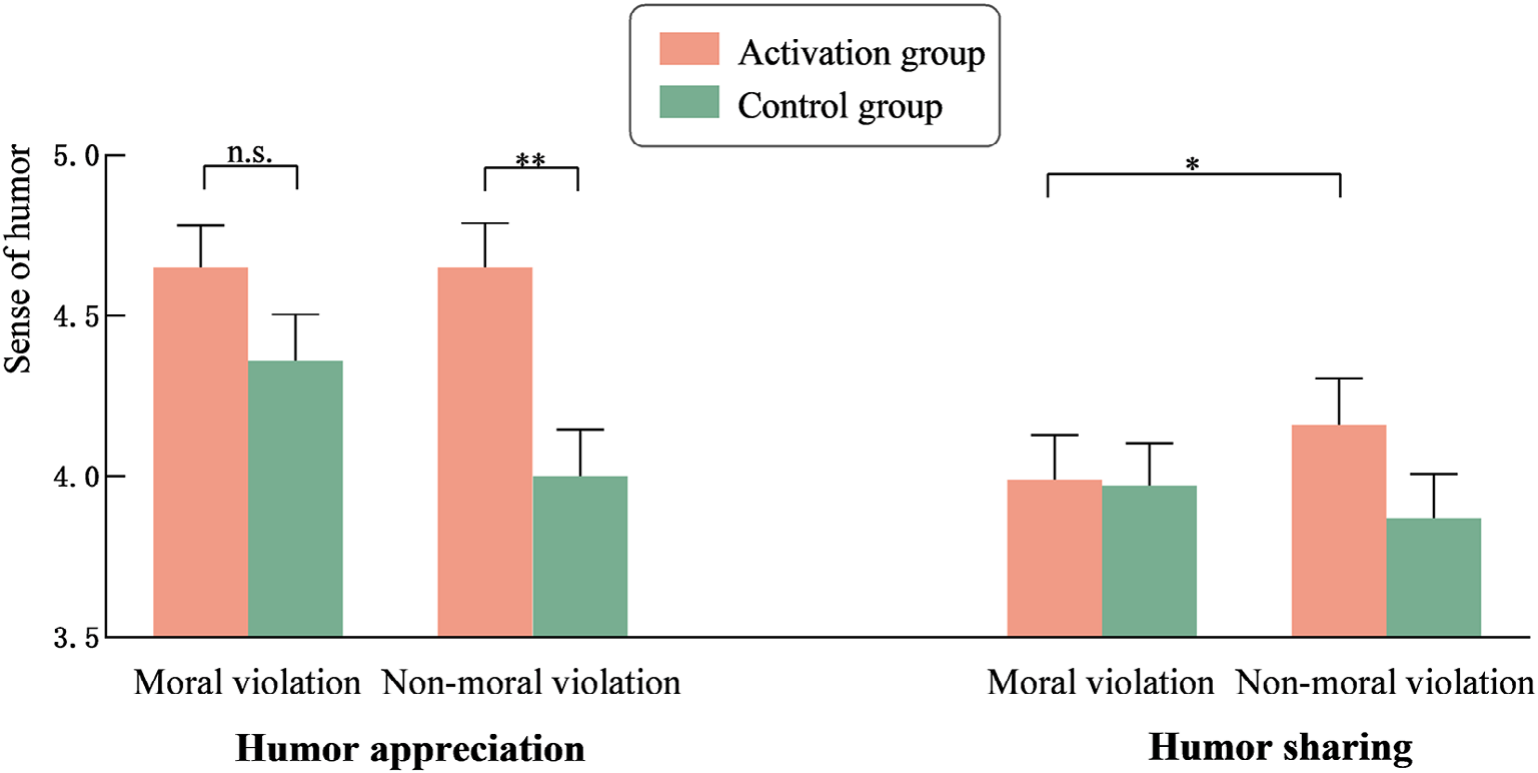
Effects of moral identity and joke type on humor appreciation and humor sharing (Study 2). * *p* < .05; **p < .01.

A 2 (moral identity: activation, control) × 2 (joke type: moral violation, nonmoral violation) repeated‐measures ANCOVA on humor appreciation was conducted, with gender and age included as covariates. The main effect of moral identity was significant, with the moral identity activation group (*M* = 4.65, *SD* = 1.18) scoring higher on humor appreciation than the control group (*M* = 4.18, *SD* = 1.31), *F* = 5.51, *p* = .020, *η*_p_^2^ = 0.03. The main effect of joke type was found to be nonsignificant, with no significant difference in humor appreciation scores between moral violation jokes (*M* = 4.50, *SD* = 1.29) and those with nonmoral violation (*M* = 4.32, *SD* = 1.35), *F* = 0.01, *p* = .935, *η*_p_^2^ = 0.00, and the results showed a significant interaction effect, *F* = 10.78, *p* = .001, *η*_p_^2^ = 0.06. Simple effects analyses revealed (see Figure 2) that there was no significant difference between the moral identity activation group (*M* = 4.65, *SD* = 1.22) and the control group (*M* = 4.36, *SD* = 1.35) for moral violation jokes, *F* = 1.77, *p* = .185, *η*_p_^2^ = 0.01, but the moral identity activation group (*M* = 4.65, *SD* = 1.27) showed a significantly higher appreciation of humor in nonmoral violation jokes than the control group (*M* = 4.00, *SD* = 1.36), *F* = 9.87, *p* = .002, *η*_p_^2^ = 0.06.

A 2 (moral identity: activation, control) × 2 (joke type: moral violation, nonmoral violation) repeated‐measures ANCOVA on humor sharing was conducted, with gender and age included as covariates. The main effects of both moral identity and joke type were found to be nonsignificant, *F*s ≤0.04, *p*s ≥ .365. However, the results showed a significant interaction effect, *F* = 4.98, *p* = .027, *η*_p_^2^ = 0.03. Simple effects analyses revealed (see Figure 2) no significant differences in humor sharing between the moral identity activation group and the control group for either moral violation or nonmoral violation jokes, *Fs* ≤0.04, *ps* ≥ .135. However, in the moral identity activation group, humor sharing was significantly lower for moral violation jokes (*M* = 3.99, *SD* = 1.27) than for nonmoral violation jokes (*M* = 4.16, *SD* = 1.33), *F* = 4.19, *p* = .042, *η*_p_^2^ = 0.02. In contrast, in the control group, humor sharing for moral violation jokes (*M* = 3.97, *SD* = 1.25) was not significantly different from that for nonmoral violation jokes (*M* = 3.87, *SD* = 1.26), *F* = 1.30, *p* = .256, *η*_p_^2^ = 0.01.

Study 2 explored the moderating effects of joke type on the relationship between moral identity and humor appreciation and sharing. Unlike the findings of Study 1, Study 2 did not find that moral identity reduced individuals' humor appreciation and sharing for jokes involving moral violations. The combined results from Studies 1 and 2 necessitate further exploration into why manipulated moral identity did not significantly impact humor appreciation and sharing under the condition of moral violation jokes. It is crucial to examine whether the effect is due to the influence of a distant social distance on the processing of moral violation information by activated moral identity, leading to the identification of moral violation jokes as benign. To address these questions, Study 3 expanded the research by incorporating variables for social distance and benign judgment (CHB) in the condition of moral violation jokes.

## STUDY 3

### Method

#### Design and participants

Study 3 employed a 2 × 2 between‐subjects design, with independent variables being moral identity (activation, control) and social distance (close, distant). The mediating variable was benign judgment (CHB), and the dependent variables were humor appreciation and humor sharing.

Using G*Power 3.1 (Faul et al., 2007), and assuming the effect size = 0.25, alpha = 0.05, power = 0.80, we calculated that a minimum sample size of 128 participants was necessary to adequately detect the anticipated effects. A total of 202 college students were recruited to participate in Study 3. After excluding 30 participants who either failed the recollection and writing tasks, did not maintain a serious attitude during the experiment, or did not complete the experimental process, 172 participants (93 females, 79 males; age range 17–29; *M*_age_ = 20.89, *SD*_age_ = 2.11) were included in the final analysis. Participants were promised that all information collected would be used solely for research purposes. Upon completion of the study, each participant received a payment of 10 RMB.

#### Material and procedure

##### Basic personal information and manipulation of moral identity

The collection of basic personal information and manipulation of moral identity in Study 3 were generally consistent with the methods used in Study 2. The difference is that in the basic information collection section, participants had been randomly assigned to either the close or distant social distance condition. In the close condition, participants were instructed to write down the name of a person with whom they had a close relationship, whereas the distant condition did not include this requirement.

##### The manipulation of social distance

Five jokes involving moral violations that could manipulate social distance were selected from the material in Study 2. In the close condition, the target individual in the jokes presented to participants was the name of a close person the participant had previously written about; in the distant condition, participants saw the target of the jokes referred to by a generic name, “someone.” An examination of the operational validity of social distance was tested by *Inclusion of Other in the Self Scale* (Aron et al., 1992; Zi & He, 2019). Participants were then presented with seven pairs of concentric circles, with the numerical value of the choice indicating the degree of overlap between the circles, representing the closeness of the target to the participant's social circle—the higher the numerical choice, the greater the overlap.

##### Measurement of humor appreciation, sharing, and benign judgment

In Study 3, the focus continued to be on participants' humor appreciation and humor sharing. Building on the high accuracy of humor comprehension demonstrated by all participants in Study 2, humor comprehension was not reassessed in Study 3.

At the end of the study, the CHB scale was introduced to evaluate participants' benign judgments regarding jokes (Hodson et al., 2010). The scale consists of six items, the sixth of which is reverse scored, and this study used a 7‐point scale ranging from 1 (*completely disagree*) to 7 (*completely agree*), with higher scores representing a stronger endorsement of the beliefs that “jokes are just jokes.”

The rest of the experiment remained consistent with Study 2.

### Results

#### The validity tests of the manipulation of moral identity

First, coded data were analyzed for five coders. According to the first item, Kendall's *W* for the coders' ratings was 0.87 (*p* < .001), indicating strong reliability among the coders. Moreover, the coders identified 20 participants in the control group whose recollections involved moral behaviors and 10 participants in the activation group whose recollections were not morally related. Consequently, the data from these 30 participants were excluded from subsequent analyses.

An independent samples *t‐*test based on the coders' ratings of the second item showed that the activation group (*M* = 5.36, *SD* = .42) had significantly higher moral scores than the control group (*M* = 4.07, *SD* = .24), *t* = 24.73, *p* < .001, Cohen's *d* = 3.77. Subsequently, an independent samples *t*‐test was conducted using the self‐rated counts of the 172 participants as the dependent variable. The results indicated that the activation group (*M* = 6.24, *SD* = 0.62) had significantly higher moral scores than the control group (*M* = 4.94, *SD* = 1.38), *t* = 7.96, *p* < .001, Cohen's *d* = 1.22. These findings suggest that the manipulation of moral identity was effective.

#### The validity tests of the manipulation of social distance

An independent samples *t‐*test was employed to analyze the perceived overlap between conditions. The results indicated that the perceived overlap was significantly greater in the close condition (*M* = 5.55, *SD* = 1.37) than in the distant condition (*M* = 2.93, *SD* = 1.55), *t* = 11.70, *p* < .001, Cohen's *d* = 1.79. This finding suggests that the manipulation of social distance was effective.

#### The effect of moral identity and social distance on humor appreciation and sharing

**FIGURE 3:**
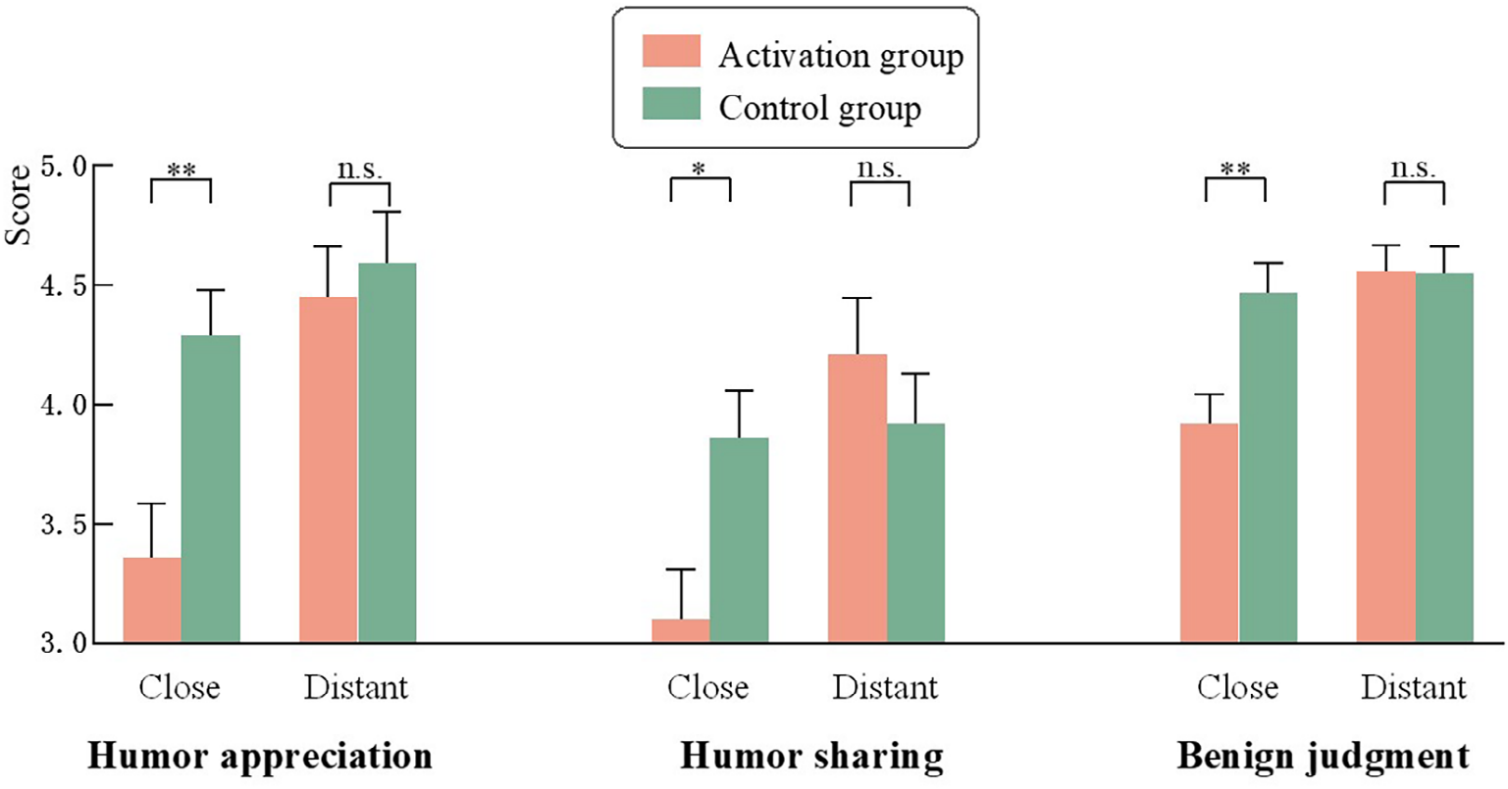
Effects of moral identity and social distance on humor appreciation, humor sharing, and benign judgment (Study 3) (error lines are standard errors). * *p *< .05; ** *p* < .01.

A 2 (moral identity: activation, control) × 2 (social distance: close, distant) between‐subjects ANCOVA on humor appreciation was conducted, with gender and age included as covariates. The main effect of moral identity was found to be significant, with the moral identity activation group (*M* = 3.78, *SD* = 1.59) exhibiting higher humor appreciation scores than the control group (*M* = 4.43, *SD* = 1.35), *F* = 6.20, *p* = .014, *η*_p_^2^ = 0.04. The main effect of social distance was also significant, with jokes presented under the close condition (*M* = 3.80, *SD* = 1.51) receiving significantly lower humor appreciation scores than those presented under the distant condition (*M* = 4.53, *SD* = 1.40), *F* = 9.26, *p* = .003, *η*_p_^2^ = 0.06. Additionally, we observed a marginal significant interaction effect, *F* = 3.17, *p* = .077, *η*_p_^2^ = 0.02. Simple effects analyses (see Figure 3) indicated no significant difference between the moral identity activation group (*M* = 4.45, *SD* = 1.39) and the control group (*M* = 4.59, *SD* = 1.42) in humor appreciation for distant condition, *F* = .22, *p* = .637, *η*_p_^2^ = 0.00, and the moral identity activation group (*M* = 3.36, *SD* = 1.57) showed significantly lower appreciation of humor in the close condition than the control group (*M* = 4.29, *SD* = 1.29), *F* = 10.55, *p* = .001, *η*_p_^2^ = 0.07.

A 2 (moral identity: activation, control) × 2 (social distance: close, distant) between‐subjects ANCOVA on humor sharing was conducted, with gender and age included as covariates. The main effect of moral identity was not significant, and there was no significant difference in humor sharing between the moral identity activation group (*M* = 3.53, *SD* = 1.56) and the control group (*M* = 3.89, *SD* = 1.37)，*F* = 1.39, *p* = .240, *η*_p_^2^ = 0.01. The main effect of social distance was significant, with participants showing a significantly lower willingness to share jokes presented about the close (*M* = 3.46, *SD* = 1.48) than those about distant others (*M* = 4.04, *SD* = 1.41)，*F* = 6.29, *p* = .013, *η*_p_^2^ = 0.04. Additionally, we observed a significant interaction effect, *F* = 5.08, *p* = .026, *η*_p_^2^ = 0.04. Simple effects analyses (see Figure 3) indicated no significant difference between the moral identity activation group (*M* = 4.21, *SD* = 1.45) and the control group (*M* = 3.92, *SD* = 1.39) in the distant condition, *F* = 0.50, *p* = .479, *η*_p_^2^ = 0.00. Moreover, in the close condition, the moral identity activation group (*M* = 3.10, *SD* = 1.49) exhibited significantly lower humor sharing than the control group (*M* = 3.86, *SD* = 1.38), *F* = 6.82, *p* = .010, *η*_p_^2^ = 0.05.

#### The mediating role of benign judgment

A 2 (moral identity: activation, control) × 2 (social distance: close, distant) between‐subjects ANCOVA on benign judgment (CHB) was conducted, with gender and age included as covariates. The main effect of moral identity was found to be significant, with the moral identity activation group (*M* = 4.17, *SD* = 0.86) exhibiting lower benign judgment scores than the control group (*M* = 4.51, *SD* = 0.67), *F* = 5.16, *p* = .025, *η*_p_^2^ = 0.04. The main effect of social distance was also significant, with the close condition (*M* = 4.18, *SD* = .83) showing lower benign judgment scores than the distant condition (*M* = 4.56, *SD* = 0.67), *F* = 8.56, *p* = .004, *η*_p_^2^ = 0.06. Additionally, we observed a significant interaction effect, *F* = 4.94, *p* = .028, *η*_p_^2^ = 0.03. Simple effects analyses (see Figure 3) indicated no significant difference between the moral identity activation group (*M* = 4.56, *SD* = 0.66) and the control group (*M* = 4.55, *SD* = 0.69) on benign judgment in the distant condition, *F* = 0.00, *p* = .972, *η*_p_^2^ = 0.00. In the close condition, the moral identity activation group (*M* = 3.92, *SD* = 0.88) showed significantly lower benign judgment than the control group (*M* = 4.47, *SD* = 0.66), *F* = 11.69, *p* = .001, *η*_p_^2^ = 0.08.

**FIGURE 4:**
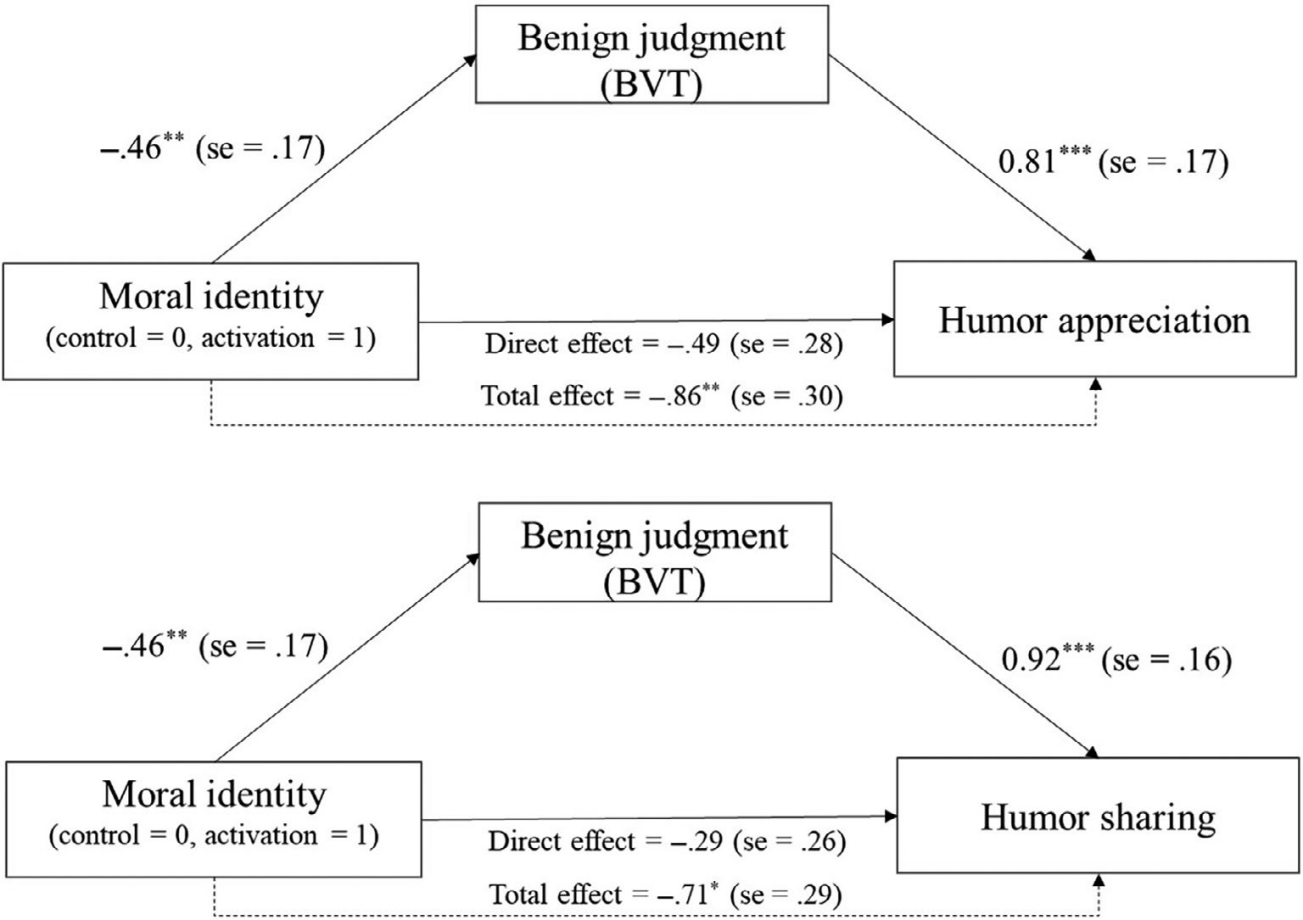
Indirect effect of condition on humor appreciation, humor sharing via benign judgment (unstandardized coefficients). ** *p* < .01; ***p < .001.

Mediation analyses were conducted using Model 4 of the Process macros for SPSS (Hayes, 2013). The results showed that in the close condition, benign judgments mediated the relationship between moral identity and both humor appreciation and humor sharing (see Figure 4). However, in the distant condition, the mediation model did not hold, *B*_appreciation_ = 0.02, *SE* = 0.30, *p* = .940, 95% CI = [−0.58, 0.63]; *B*_sharing_ = 0.41, *SE* = 0.31, *p* = .190, 95% CI = [−0.21, 1.04].

## DISCUSSION

Drawing upon the BVT, the present research aimed to address the inconsistent findings from previous studies regarding the impact of moral identity on individuals' sense of humor, and employs a three‐study design to investigate this issue. Consistent with our predictions, moral identity does not necessarily diminish the sense of humor. Specifically, humor type (moral violation, nonmoral violation) and social distance (close, distant) emerged as important moderating variables influencing the relationship between moral identity and individuals' sense of humor. When jokes did not involve moral violation, moral identity was found to have a positive impact on one's sense of humor, including both humor appreciation and sharing. However, when jokes contained moral violations, moral identity exerted a dampening effect on one's sense of humor, particularly when the jokes targeted individuals within a close social distance.

As we hypothesized, both Study 1 and Study 2 demonstrated that moral identity is positively associated with a sense of humor in adaptive humor (and nonmoral violation jokes). Although our findings differ from some previous research, which indicated no significant difference in the degree of humor appreciation for nonmorally violating jokes across different levels of moral identity (Yam et al., 2019), we believe the relationship between moral identity and sense of humor is complex. Both moral identity and humor encompass positive and negative aspects. The BVT suggests that if an individual judges the content of a violation as harmless, the basic conditions for humor are met (McGraw & Warren, 2010). Individuals with a heightened moral identity tend to engage in various pro‐social behaviors (Guo et al., 2022; Patrick et al., 2018). Simultaneously, adaptive humor serves to alleviate negative emotions in others and facilitate positive emotional experiences (Kugler & Kuhbandner, 2015). Therefore, when humor excludes negative elements such as aggression and degradation, it can effectively regulate the negative emotions of others while exhibiting traits like love and helpfulness. This makes it a favorable choice for individuals possessing a strong moral identity. Furthermore, with the development of society, humor has evolved into an essential aspect of life. The use of humor is no longer considered simply “unorthodox,” but rather a manifestation of wisdom and ability (Yue, 2010; J. Liu, 2016; Cao & Hou, 2023). As Mark Twain perceived his own humorous literature: “Although our writings may appear shallow and frivolous on the surface, they serve a solemn purpose—to satirize hypocrites, expose deceitful liars, and dismantle superstitions amidst laughter—objectives that only a ‘humorist’ can accomplish” (X. Zeng, 1995). To some extent, humor also functions as an instrument for upholding justice. These findings suggest that individuals with high moral identities can employ humor as an aid in achieving their pro‐social goals.

Consistent with previous research findings, our study results indicate that the presence of moral violations in jokes hinders the positive impact of moral identity on humor appreciation and sharing. In Study 1, high levels of moral identity negatively predicted nonadaptive humor, which involves moral violations. However, Study 2 yielded different results from Study 1, showing no significant effect of moral identity on humor appreciation and sharing in the moral violation condition. According to BVT (McGraw & Warren, 2010), if the manipulation of moral identity does not yield a significant impact on sense of humor, it is plausible that participants in the activated moral identity group, similar to those in the control group, may perceive jokes involving moral violations as benign and acceptable. Consequently, there may be no significant disparity in the sense of humor between the two groups. In previous research (Yam et al., 2019), it was found that when an individual's moral identity is activated, their attention shifts toward the moral violation component of a joke. If a joke contains a moral violation, it tends to be perceived as unfunny and reduces the willingness to share it with others.

The findings of the current research do not entirely align with the study of Yam et al. (2019). Nevertheless, our results are consistent with previous research indicating a significant divergence in the comprehension of “immoral” between Eastern cultures, exemplified by China, and Western cultures (Buchtel et al., 2015; Buchtel et al., 2022). The difference lies in the perception of “immoral” in Western culture is directed toward “harmful,” whereas in Eastern culture, it is more directed toward “uncivilized.” Specifically, the jokes employed by Yam et al. encompassed a substantial number of components that posed existential threats and exhibited pronounced moral transgressions that were easily identifiable. Meanwhile, in the questionnaire of Study 1, the items of nonadaptive humor clearly highlighted the existence of moral violation nature of the humor, allowing the individuals with high levels of moral identity to effortlessly identify the moral violations. In Study 2, considering the cultural adaptability of jokes, the jokes of moral violation were chosen to have significantly lower degrees of moral violation. It is possible that individuals with high levels of moral identity recognized these jokes of the current experiment as benign. Although Study 2 did not fully support the results of Study 1, it also provided support for the notion that the presence of moral violation impedes the positive impact of moral identity on sense of humor. The results of humor sharing in Study 2 revealed that, for the moral identity activation group, their inclination to share nonmoral violation jokes was significantly higher than that for moral violation jokes. This finding suggests that the presence of a moral violation component still exerts an influence on the relationship between moral identity and humor.

The current research findings also highlight that moral identity exerts a further dampening effect on both humor appreciation and humor sharing, mediated by benign judgments, particularly when moral violation jokes are employed within close social distance. According to the construal level theory, an individual's selective attention in information processing is influenced by social distance. Greater social distance leads to a tendency for individuals to rely on more general, central, and decontextualized features when representing an event (Y. C. Li et al., 2009). Accordingly, the cognitive evaluation of jokes by individuals with moral identity may vary across different conditions of social distance. When moral violation jokes are at a distant social distance, both the moral identity activation group and the control group may exhibit heightened attention toward the unexpected element of the joke due to the relatively mild severity of the moral violation, thereby facilitating a benign disregard for potential offense caused by such jokes. In contrast, when a moral violation joke occurs at a close social distance, individuals devote more attention to the target of the joke, the more likely the individuals perceive the content of the joke to occur, and the more threatening the content becomes (McGraw et al., 2012). Consequently, jokes involving close social distance are not perceived as purely humorous. Simultaneously, under conditions of close social distance, individuals with elevated levels of moral identity are prompted to engage in heightened moral processing. They are more inclined to recognize that the individual targeted in the joke may feel offended and perceive the joke as non‐benign. This recognition leads to diminished appreciation for and a reduced inclination to share humor derived from such jokes.

In terms of theoretical significance, this research has broadened the scope of cultural applicability of BVT. Originally proposed by Western scholars, BVT's applicability in the Chinese cultural context, given the substantial differences in humor culture between China and the West, needed to be explored.

This research utilized Chinese jokes as experimental stimuli to investigate the relationship between moral identity and humor. The experimental results support the cross‐cultural application of BVT, suggesting that the BVT, as a newly proposed humor theory in recent years, can break through the cultural gap and be applicable to both Chinese and Western humor research. Methodologically, this study employed a combination of questionnaires and behavioral experiments to examine the relationship between moral identity and sense of humor. The findings demonstrated a relatively stable consistency, addressing to some extent the issue of conflicting results in previous studies and contributing to the understanding of how moral identity impacts sense of humor. Additionally, this study has practical implications. The findings can be directly applied to interpersonal interaction. The study suggests that individuals can actively incorporate nonmoral violation humor into their interactions but should exercise caution when using moral violation humor, particularly when engaging with individuals who possess high levels of moral identity.

The present research, however, has certain limitations that necessitate further investigation in subsequent research. Firstly, it is crucial to acknowledge that humor takes many forms beyond jokes, and a comprehensive understanding of the sense of humor requires consideration of these diverse forms. As a common form of humor, jokes are contextual and easily manipulated, which is why Studies 2 and 3 in this research selected jokes as humorous materials. This selection aimed to explore the relationship between moral identity and sense of humor more deeply from an experimental perspective, building on references to previous studies. On this basis, future research may consider distinguishing between different styles of humor—affiliative, self‐enhancing, aggressive, and self‐defeating (as categorized by Martin et al., 2003)—to further examine the relationship between moral identity and sense of humor, thereby complementing the existing body of research on humor.

Additionally, the range of social groups to which the results of this research apply is limited. Research has shown that as age increases, there is a trend of decreasing sense of humor, particularly for moral violation jokes, with younger individuals finding them the most humorous, followed by middle‐aged individuals, and older individuals showing the least appreciation of humor (Stanley et al., 2014). This research primarily focused on college students and did not take into account the role of age. Future research can expand the research population to verify the practicality and stability of the findings of this study.

## CONCLUSION

This research examined the effect of moral identity on sense of humor and sought to elucidate its underlying psychological mechanisms. The following conclusions were reached:

1. High levels of moral identity, or its activation, can significantly and positively predict aspects of sense of humor, including humor appreciation and sharing.
2. When a moral violation in humor targeting individuals with close social distance, the activation of moral identity leads to a decrease in appreciation and sharing of such humor, where benign judgment plays a mediating role.

## FUNDING INFORMATION

This work was supported by the Major Support Projects for Emerging (Interdisciplinary) Disciplines of Philosophy and Social Sciences of Zhejiang Province in China (Grant number: 19XXJC04ZD‐2).

## CONFLICT OF INTEREST STATEMENT

The authors declare there are no conflicts of interest.

## ETHICS STATEMENT

This research was conducted in accordance with the Declaration of Helsinki. The Ethics Committee of School of Psychology, Zhejiang Normal University, approved the study. The participants interested in participating read the informed consent which contained the objectives, benefits, and risks of participation; those who agreed to participate in the study gave their informed consent, accepting to continue with the survey. The participants were informed that their responses to task would be anonymous and confidential, and the data collected would be used for academic research only.
